# Supplementary material for: Public Awareness and Risk Perceptions of Endocrine Disrupting Chemicals: A Qualitative Study
Source: Int J Environ Res Public Health. 2020 Oct 23;17(21):7778. doi: 10.3390/ijerph17217778 (PMC7660691; doi:10.3390/ijerph17217778)
Supplement: Supplementary file 1 [file ijerph-17-07778-s001.pdf]

International Journal of
Environmental Research
and Public Health

MDPI

Study on Chemicals in Food and the Environment

Participant Packet

February, 2020
Institute for Global Food Security
School of Biological Sciences
Queens University Belfast

Facilitators: Melissa Kelly PhD student

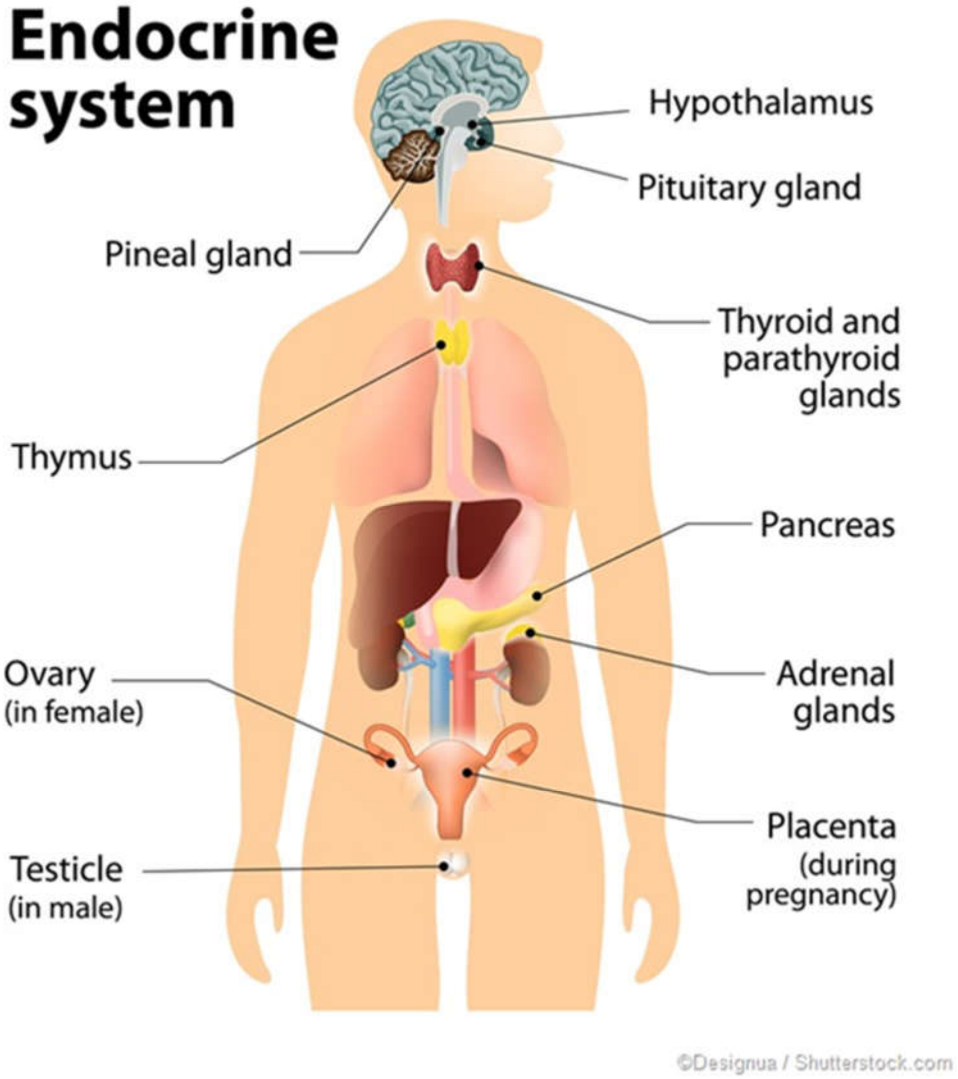

Figure 1. Diagram of the Human Endocrine System.

The endocrine system is made up of 8 major glands in your body, such as your thyroid and your reproductive organs. These glands send out signals, known as hormones ("chemical messengers") into the bloodstream. Hormones communicate between different organs in the body.

Endocrine disrupting chemicals are naturally occurring or manmade substances that interfere with the way that these hormones work in the body by mimicking or blocking their target receptors.

Table 1. Endocrine Disrupting Chemicals.

**Endocrine Disrupting Chemical/Group.**
Bisphenol A (BPA)
Phthalates
Pesticides
Dioxins
Mycotoxins
Brominated Flame Retardants
Phytoestrogen

Table 2. Sources of Endocrine Disrupting Chemicals

**Endocrine Disrupting Chemical /Group**	**Source**
BPA	plastic food/beverage containers,<br>canned foods<br>plastic bags
Phthalates	plastic containers<br>Dust/indoor air<br>personal care products
Pesticides	fruit and veg<br>soil<br>meats
Dioxins	soil<br>dairy foods<br>meat and fish<br>waste sites
Mycotoxins	animal feed<br>soil<br>grains<br>seeds and nuts
Brominated Flame Retardants	textiles<br>furniture<br>electronic castings<br>circuitry
Phytoestrogen	soy beans<br>cabbage<br>sesame seeds

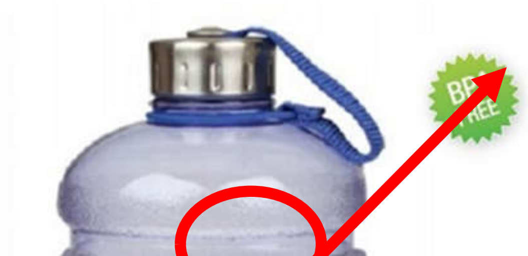

Figure 2. BPA Free Water Bottle.

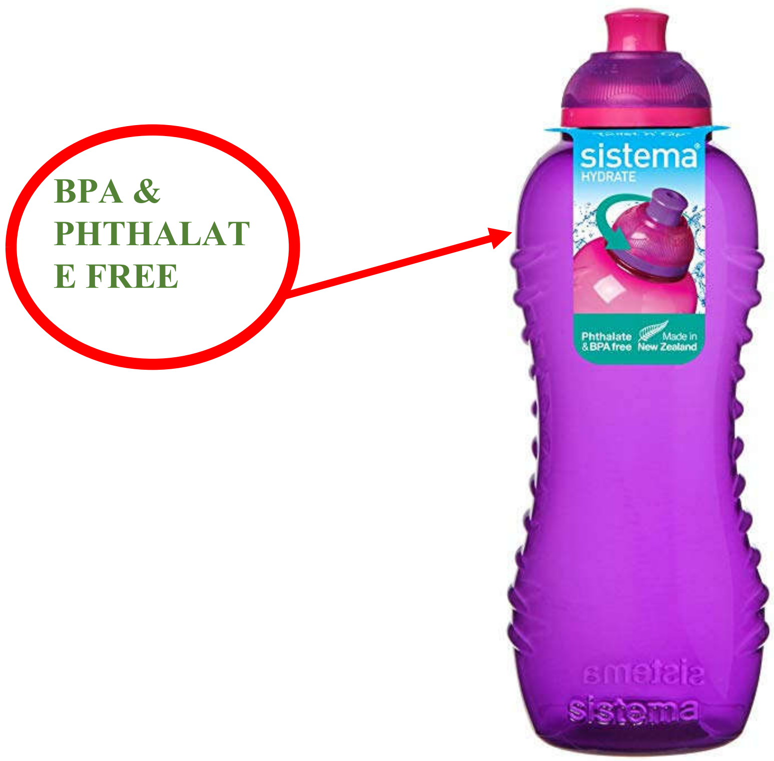

Figure 3. BPA and Phthalate Free Water Bottle.

Suggestions to Reduce Exposure

Endocrine Society Org

Read the labels. On plastic bottles, a #1, #2, or #4 in the recycling sign means that the product is free of BPA, a still commonly-used EDC. Labels for cleaning supplies, facial washes, and detergents also sometimes indicate the presence or absence of some EDCs known to be a potential risk, such as phthalates.
Keep it fresh. Minimize consumption of processed/canned foods as much as possible, and use filtered as opposed to bottled water.
Watch out for leaching. Avoid storing canned or plastic-packaged foods in hot areas, like the boot of a car on a summer day. Also, avoid microwaving or heating food in plastic containers, as EDCs could leach out from the container and into your food and body.
Reduce pesticide use. For produce, wash fresh fruit and vegetables with tap water to remove chemicals.

Information on Endocrine Disrupting Chemicals

"EDCs have the capacity to interfere with tissue and organ development and function, and therefore they may alter susceptibility to different types of diseases throughout life. This is a global threat that needs to be resolved.
Humans and wildlife are exposed to multiple EDCs at the same time, and there is justifiable concern that different EDCs can act together and result in an increased risk of adverse effects on human and wildlife health.
New sources of exposure to EDCs, in addition to food, have been identified and include indoor environments and electronics recycling and dumpsites (the latter being issues of particular concern for developing countries and countries with economics in transition). Children can have higher exposures due to their hand to-mouth activities and higher metabolic rate.
Male and female reproductive health, thyroid-related disorders, hormone-related cancers, metabolic disorders (obesity, diabetes), and developmental disorders." World Health Organization, 2012.

Websites for more information:

World Health Organization: https://www.who.int/ceh/publications/endocrine/en/
Breast Cancer UK: https://www.breastcanceruk.org.uk/science-and-research/background-briefings/endocrine-disrupting-chemicals/
Environmental Protection Agency: https://www.epa.gov/endocrine-disruption
Natural Resources Defense Council: https://www.nrdc.org/stories/9-ways-avoid-hormone-disrupting-chemicals

PROTECTED website: http://protected.eu.com/index.html
Twitter: @PROTECTED_ITN
Facebook: https://www.facebook.com/PROTECTED.ITN
